# Personalized PHQ-9 test length using probability density estimation based on conditional probability and K-Nearest Neighbours

**DOI:** 10.1016/j.invent.2026.100919

**Published:** 2026-02-12

**Authors:** Zahraa Abdulhussein, Marcia Scazufca, Pepijn van de Ven

**Affiliations:** a Department of Electronic and Computer Engineering, University of Limerick, Ireland; b Hospital das Clinicas HCFMUSP, Faculdade de Medicina, Universidade de Sao Paulo, Sao Paulo, Brazil

**Keywords:** Depression, PHQ-9, PHQ-DEP-4, PHQ-2, Dynamic tests, Adaptive tests, Conditional probability, K-Nearest Neighbour, KNN

## Abstract

The Patient Health Questionnaire-9 (PHQ-9) is a tool consisting of nine items designed to assess the severity of depression in individuals. Shorter versions have been developed such as the PHQ-DEP-4, which includes four items, and the PHQ-2, which consists of just two. These fixed-length formats have been developed to facilitate rapid screening, particularly for identifying individuals eligible for clinical trials. In this study, we propose and evaluate a dynamic version of the PHQ-9, in which the number of questions administered varies according to the respondent’s answers. This adaptive approach estimates the likelihood of depression conditional on the responses given thus far and can terminate the assessment early when a confident classification (depressed or non-depressed) can be made before all nine questions are completed. The model relies on a historical datasets of completed PHQ-9 interviews to inform these decisions. When a matching response pattern is not available in the historical data, a K-Nearest Neighbours (KNN) model is applied to estimate the probability density for this pattern. Experimental results demonstrate that the dynamic PHQ-9 model outperforms the PHQ-DEP-4, achieving higher sensitivity, specificity, and Youden index, while also reducing respondent burden by requiring fewer questions on average.

## Highlights

• Developed a dynamic PHQ-9 model that adapts the number of questions based on responses.
• Uses historical data, conditional probability and KNN to estimate depression probability for early classification.
• Outperforms PHQ-DEP-4 with higher sensitivity, specificity, and Youden index.
• Reduces respondent burden: 47%–66% of respondents required only 2 questions.
• Demonstrates robust performance across diverse populations with varying depression prevalence.

## 1 Introduction

The Patient Health Questionnaire-9 (PHQ-9) is a screening questionnaire for assessing the severity of depression. Developed in 2001 (Kroenke and Spitzer, 2002), the PHQ-9 consists of nine items, each targeting a specific depressive symptom. Responses are recorded on an ordinal scale ranging from 0 to 3, reflecting increasing symptom severity (see Table 1 for the full list of items). In the original validation study (Kroenke et al., 2001), the authors demonstrated that a total score of 10 or higher indicates probable major depressive disorder. However, the original work primarily focused on cross-sectional diagnostic accuracy and did not extensively examine the stability or validity of PHQ-9 scores over time, particularly following treatment. Subsequent research has identified limitations in the use of the PHQ-9 total score as a sole indicator of depression severity, particularly when used to monitor changes over time. These findings suggest that summed scores may not always fully reflect changes in individual symptom profiles, motivating ongoing efforts to improve the efficiency and adaptability of depression screening tools (Hlynsson et al., 2025, Kristófersdóttir et al., 2024). Importantly, these limitations do not negate the practical utility of the PHQ-9 as a screening instrument; rather, they highlight the need for more flexible and efficient methods of administering established questionnaires that are already widely used in clinical and research settings, particularly in contexts where time and respondent burden are critical considerations.

In screening contexts, shorter assessments are especially desirable, as longer questionnaires increase respondent burden, require more administration time, and may negatively affect completion rates and data quality. Shortened versions of the questionnaire, such as the PHQ-2 and PHQ-DEP-4, have been developed to enable rapid pre-screening prior to more comprehensive clinical assessment (Ishihara et al., 2019, Kroenke et al., 2003). While these abbreviated tools reduce respondent burden, they retain a fixed item structure and do not adapt to individual response patterns. A limitation shared by existing fixed length screening instruments is their inability to tailor the assessment process based on the information already provided by the respondent. There may therefore be benefit in dynamically determining whether additional questions are necessary, based on earlier responses. For example, some individuals may provide sufficient information after only two items, whereas others may require additional items to achieve an adequate level of precision, thereby minimizing assessment length while maintaining informational sufficiency.

In our previous work (Abdulhussein and van de Ven, 2024), we addressed this gap by introducing a dynamic screening approach that adjusts the number of administered items according to an individual’s response pattern. The proposed model aimed to maintain an acceptable level of measurement precision while reducing respondent burden. In this framework, items are administered sequentially, and each response is compared against an estimated lower threshold and a fixed upper threshold. Lower thresholds were estimated using a grid search method applied across the entire dataset. In the present study, we extend this approach by replacing grid search with probability density estimation (PDE) based on conditional probability and k-nearest neighbours (KNN) methods to estimate both lower and upper thresholds. This statistical framework enables data-driven estimation, avoids exhaustive search, and improves computational efficiency and scalability. Furthermore, we introduce a personalization component: instead of estimating a single set of optimal thresholds for the entire dataset, we estimate individualized upper and lower thresholds for each respondent. This modification aims to increase adaptivity and better accommodate inter-individual differences in response patterns.

**Table 1:** PHQ-9 Questions.

Over the last 2 weeks, how often have you been bothered by the following problems:
phq1: Little interest in doing things?
phq2: Feeling down, depressed, or hopeless?
phq3: Trouble sleeping or sleeping too much?
phq4: Feeling tired or having little energy?
phq5: Poor appetite or overeating?
phq6: Feeling bad about yourself?
phq7: Trouble concentrating on things?
phq8: Moving or speaking slowly or too fast?
phq9: Thought you would be better off dead?

## 2 Related works

Whilst the PHQ-9 is a popular screening instrument for depression, efforts have been undertaken to reduce interviewee burden by reducing the number of questions asked. Researchers (Ishihara et al., 2019) developed the aforementioned four-item version of the PHQ-9, known as the PHQ-Dep-4, using optimal test assembly (OTA) methods with item response theory. This approach aimed to maintain similar performance to the PHQ-9 while reducing the number of items. This abbreviated test consists of questions phq1, phq2, phq6, and phq8. In the original study, the authors used data from 7850 participants across 20 primary studies conducted in multiple countries with the PHQ-9 completed in English. Using a cutoff ≥10 for the PHQ-9 and evaluating various cutoffs for the PHQ-DEP-4, the authors found the optimal cutoff for the latter to be ≥4. The PHQ-Dep-4 demonstrated a Cronbach’s alpha of 0.805, indicating acceptable internal consistency. Its sensitivity and specificity were 0.788 and 0.837, respectively, closely aligning with the full PHQ-9, which had sensitivity and specificity of 0.761 and 0.866, respectively. The high correlation (r = 0.919) between the PHQ-Dep-4 and the full PHQ-9 suggests that the shorter version retains much of the original instrument’s diagnostic capability.

A meta-analysis (Harel et al., 2022) further validated the PHQ-Dep-4 by analysing a large dataset of 34,698 participants from 75 primary studies worldwide. The study compared its diagnostic accuracy to the full PHQ-9 across different types of clinical interviews, including semi-structured, fully structured, and MINI (Mini International Neuropsychiatric Interview) assessments. The optimal cutoff for PHQ-Dep-4 remained ≥4, consistent with earlier research. Sensitivity and specificity varied depending on the diagnostic interview used: for semi-structured interviews (sensitivity: 0.88, specificity: 0.79), fully structured interviews (sensitivity: 0.68, specificity: 0.85), and MINI (sensitivity: 0.80, specificity: 0.83). In comparison, the PHQ-9 demonstrated slightly higher specificity but comparable sensitivity. The results confirmed that the PHQ-Dep-4 offers a viable alternative to the full PHQ-9 for depression screening, particularly in settings where time constraints or survey fatigue are concerns.

Another widely recognized short form of the PHQ-9 is the PHQ-2, which consists of only the first two items of the PHQ-9. This ultra-brief tool serves as a pre screening measure before administering the full PHQ-9. Extensive research has validated its use (Kroenke et al., 2003, B. Löwe and Gräfe, 2005, Li et al., 2007), demonstrating its effectiveness in identifying individuals at risk for major depressive disorder. A meta-analysis (Levis et al., 2020) assessed the diagnostic accuracy of the PHQ-2 alone and in combination with the PHQ-9 using data from 44,318 participants, including 4572 with major depression. Results indicated that a PHQ-2 score ≥2, followed by a PHQ-9 score≥10, achieved a sensitivity of 0.82 and specificity of 0.87, similar to the PHQ-9 alone (sensitivity: 0.86, specificity: 0.85). This approach effectively reduced the number of participants required to complete the full PHQ-9 by 57%, making it a practical method for streamlining depression screening in large-scale assessments.

In addition to the PHQ-2 and PHQ-Dep-4, other ultra-brief screening methods have been explored to efficiently identify depression. For instance, recent research (Glavin et al., 2023) employed machine learning techniques to evaluate all 36 possible two-item combinations of the PHQ-9 to identify the most predictive pairs for pre screening depressive symptomatology. The study found that the pairing of phq2 (depressed mood) and phq4 (low energy) demonstrated higher predictive performance compared to the traditional PHQ-2. This suggests that alternative item pairings may enhance the efficiency and accuracy of depression screening tools.

## 3 Methods

This study introduces a dynamic method aimed at minimizing the number of questions required to reach a diagnostic decision. Following standard terminology in machine learning, we refer to this decision-making process as a model. The approach sequentially processes the PHQ-9 questions in their original order, evaluating responses after each question to determine whether a decision can be made. At each step, one of three possible actions is taken based on the cumulative score at that point:

1. If the cumulative score is less than or equal to an estimated lower threshold, the individual is classified as **non-depressed**.
2. If the cumulative score exceeds an estimated upper threshold, the individual is classified as **depressed**.
3. If the cumulative score falls between the two thresholds, the next question is presented, and the updated score is reassessed against updated upper and lower thresholds.

This section provides a comprehensive explanation of the proposed methodology, detailing how decisions are made dynamically and how to generate the optimal thresholds using an estimate of the probability of being deemed depressed or non-depressed conditioned on the answers tot he PHQ-9 items already provided. It also describes how a KNN model is used to provide an estimate of this probability in case the dataset does not provide the required data. Additionally, it outlines the datasets utilized to evaluate the model’s effectiveness, including their sources and key characteristics.

### 3.1 Data

#### 3.1.1 NHANES

The data was obtained from the National Health and Nutrition Examination Survey (NHANES) (for Disease Control and , CDC), a nationwide survey that assesses the health and nutritional status of adults and children in the United States. NHANES includes the PHQ-9 to evaluate depressive symptomatology. Each year, approximately 5000 individuals are selected through a randomized, scientifically rigorous process to ensure a representative sample of the diverse U.S. population. For this analysis, we used data collected from 2005 to 2020, comprising 49,461 records that include demographic information and PHQ-9 responses. Records with responses indicating refusal to answer (coded as 7) or uncertainty (coded as 9) were excluded, along with those containing missing values. After data cleaning, the final dataset consisted of 44,749 complete records. Table 2 presents the dataset’s characteristics.

**Table 2:** Characteristics of NHANES data.

Characteristics	Number (*%*)	Characteristics	Number (*%*)
Sample	44,535	Female	22,652 (50)
Age, years	48.0±18.6	Marital Status	
Ethnicity		Married	17,574 (39)
Mexican American	6853 (15)	Widowed	2695 (<1)
Other Hispanic	4278 (<1)	Divorced	3751 (<1)
Non-Hispanic White	18,114 (40)	Separated	1159 (<1)
Non-Hispanic Black	10,107 (22)	Never married	1159 (<1)
Other Race	5183 (11)	Living with partner	2842 (<1)
Education Level – Youth		Refused	19 (<1)
9th Grade	50 (<1)	Do not know	1 (<1)
10th Grade	107 (<1)	Missing	9892 (22)
11th Grade	416 (<1)	Family Income - Annual	
12th Grade, No Diploma	166 (<1)	$0 to $4999	169 (<1)
High School Graduate	735 (<1)	$5000 to $9999	254 (<1)
GED or Equivalent	47 (<1)	$10,000 to $14,999	392 (<1)
More than high school	567 (<1)	$15,000 to $19,999	364 (<1)
Less Than 9th Grade	42 (<1)	$20,000 to $24,999	373 (<1)
Missing	42,404 (95)	$25,000 to $34,999	611 (1)
Education Level – Adults 20+		$35,000 to $44,999	478 (1)
Less Than 9th Grade	3994 (<1)	$45,000 to $54,999	440 (<1)
9–11th Grade (Includes 12th grade with no diploma)	5670 (12)	$55,000 to $64,999	271 (<1)
High School Grad/GED or Equivalent	9799 (22)	$65,000 to $74,999	254 (<1)
Some College or AA degree	12,747 (28)	$75,000 and over	980 (<1)
College Graduate or above	9774 (21)	Over $20,000	67 (<1)
Refused	10 (<1)	Under $20,000	27 (<1)
Do not Know	22 (<1)	Refused	27 (<1)
Missing	2519 (<1)	Do not know	56 (<1)
PHQ-9 ≥10	3935 (8.8)	Missing	39,772 (89)

#### 3.1.2 PROACTIVE

The second dataset used in this study was derived from the PROACTIVE cluster randomized controlled trial (Scazufca et al., 2020, Scazufca et al., 2022), which investigated depressive symptoms among adults aged 60 and older living in socio-economically disadvantaged areas of Guarulhos, Brazil. Participants were recruited from 20 randomly selected primary care clinics, where eligible individuals were invited to complete a screening interview. Data collection occurred between 2019 and 2020 using dedicated Android apps to improve data collection fidelity (P. Van de Ven et al., 2019). The dataset includes demographic information along with responses to PHQ-9 items. The PROACTIVE dataset was similarly cleaned to remove missing values, resulting in a total of 4025 records. For a detailed overview of the dataset’s characteristics, refer to Table 3.

**Table 3:** Characteristics of PROACTIVE data.

Characteristics	Number (*%*)	Characteristics	Number (*%*)
Sample	4025	Female	2542 (63)
Age, years	68.35±6.74	Income	
Education		Up to 1 minimum wage	2462 (65)
1–4 years	1875 (55)	>1–2 minimum wage	813 (21)
5–8 years	895 (26)	>2–3 minimum wage	303 (<1)
>8 years	627 (18)	>3 minimum wage	211 (<1)
PHQ-9 ≥10	1216 (30)		

#### 3.1.3 Leave-one-out approach for preventing data leakage

In typical machine learning workflows, datasets are split into training and testing subsets to prevent over fitting and avoid data leakage. However, we did not adopt this standard train/test split approach. We instead used a leave-one-out strategy: the individual for whom the threshold was being estimated was excluded from the dataset during the computation. The remaining data was then used iteratively to generate personalized thresholds for each respondent. This approach is appropriate for our context because the model does not involve training a complex algorithm with numerous parameters, as seen in supervised machine learning. Instead, our method relies on direct probability computations or simple distance-based logic (e.g., KNN), which do not require parameter learning in the traditional sense. By excluding the individual during threshold estimation, we effectively avoid bias and ensure the model generalizes appropriately, while still preserving the full dataset for probabilistic analysis.

### 3.2 The dynamic questionnaire model

**Fig. 1:**
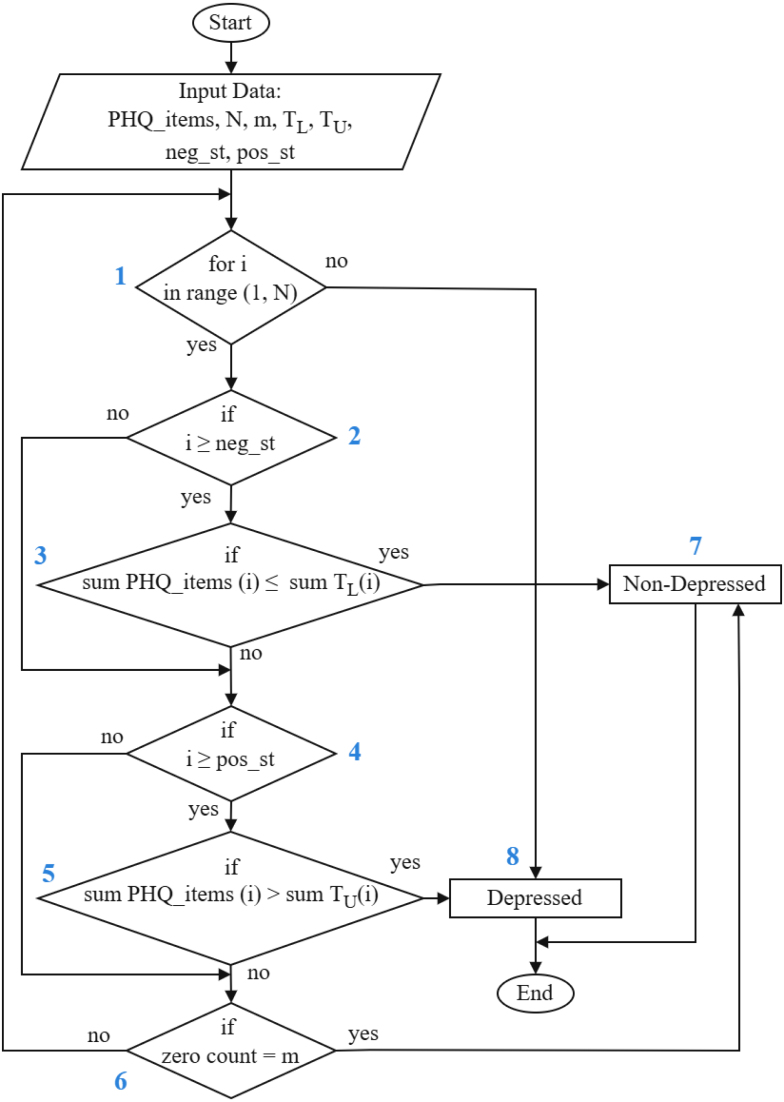
Dynamic Questionnaire Model Flow Chart.

The model operates through an iterative process, considering one PHQ-9 item at a time, as illustrated in the flow chart (Fig. 1). The two primary conditions assess whether a response meets predefined lower and upper threshold criteria, represented by the diamond shapes numbered 3 and 5 respectively in the flow chart.

Specifically, the model calculates the cumulative sum of an individual’s responses, denoted as (sum_PHQ_items(i)), and compares it to the lower cumulative threshold ($T_{L}$). If the cumulative score is less than or equal to the cumulative $T_{L}$, the respondent is classified as non-depressed, and no further questions are asked.

If this condition is not met, the model checks whether the cumulative score exceeds the upper cumulative threshold ($T_{U}$). If so, the respondent is classified as depressed, and no further questions are asked. If neither classification is reached, the model continues evaluating responses until all PHQ-9 items have been processed. Any respondent who remains unclassified after answering all questions is labelled as depressed.

For example, suppose a respondent answers 1 to phq1. At this stage, assume the $T_{L}$ is 0 and the $T_{U}$ is 2. Since the respondent’s cumulative sum score (1) is neither less than or equal to the $T_{L}$ (0) nor greater than to the $T_{U}$ (2), no classification is made, and the model proceeds to the next question, phq2. The respondent then answers 1 to phq2 as well. At this point, assume the $T_{L}$ for phq2 is 2 and the $T_{U}$ is 3. The model calculates the cumulative sums: the total response score becomes 1+1=2, the cumulative lower threshold becomes 0+2=2, and the cumulative upper threshold becomes 2+3=5. The model first compares the total response score to the cumulative lower threshold. Since 2≤2, the model classifies the respondent as non-depressed and terminates the screening process.

An additional condition, represented by diamond shape numbered 6 in the flowchart, tracks the number of zero responses. If a respondent exceeds a predefined count of zeros, they are classified as non-depressed. This condition identifies individuals who consistently answer with zeros, an early indicator of a lack of depressive symptoms, allowing the model to classify them efficiently.

The remaining conditions, represented by the diamond shapes numbered 2 and 4, determine the starting point for classification. These conditions help optimize model performance by identifying the most effective point to initiate classification. For instance, if classification of non-depressed cases is set to begin at phq2, the model does not evaluate the condition in the third diamond shape until the respondent reaches question 2. At that point, the second diamond condition is triggered, and classification begins at the third diamond. A similar process applies to positive cases, as reflected in the diamond shapes numbered 4 and 5.

#### 3.2.1 Thresholds estimation for phq2 to phq9

Our approach employs a data-driven method to estimate individualized thresholds using the datasets. For binary classification, we follow the widely accepted criterion of using a PHQ-9 total score of ≥10 to distinguish between depressed and non-depressed individuals, ensuring consistency with established research. We applied a probability density based approach to estimate these thresholds. For questions beyond phq1, the $T_{L}$ is estimated using conditional cumulative probabilities. This approach aims to classify individuals as non-depressed as early as possible. In addition, it ensures to include only individuals whose responses to previous PHQ items match those of the individual being classified, which allows for more personalized threshold estimation. We begin by calculating the conditional cumulative probability that a respondent is depressed (D), given their response history ($H=\left(r_{1},r_{2},\ldots ,r_{i}\right)$) up to the previous question (i). For the next PHQ question ($phq_{i+1}$), this probability is calculated as:

(1) $$P\left(D,phq_{i+1}\leq r|H\right)=\frac{P\left(D,phq_{i+1}\leq r,H\right)}{P\left(H\right)}$$

where:

• $P\left(D,phq_{i+1}\leq r|H\right)$ is the conditional cumulative probability of being depressed and responding with a value less than or equal to r on $phq_{i+1}$, given the history H.
• $P\left(D,phq_{i+1}\leq r,H\right)$ is the joint probability of being depressed, giving a response ≤r and having a history H.
• P(H) is the probability of the observed response history H.

Since $phq_{i+1}$ can take one of four values (r∈{0,1,2,3}), this process generates a cumulative discrete probability distribution with 4 values. These probabilities are then compared to a predefined loss parameter (λ), which represents the acceptable risk of misclassifying a depressed individual as non-depressed. The latent $T_{L}$ is then selected according to the following decision rule:

$$T_{L}=\min\left\{\begin{array}{cc} 0\; & \text{if}P\left(D,phq_{i+1}\leq 0|H\right)>\lambda \\ 1\; & \text{if}P\left(D,phq_{i+1}\leq 1|H\right)>\lambda \\ 2\; & \text{if}P\left(D,phq_{i+1}\leq 2|H\right)>\lambda \\ 3\; & \text{if}P\left(D,phq_{i+1}\leq 3|H\right)>\lambda \;\;\text{or}\;P\left(D|H\right)=0 \end{array}\right)$$

To estimate the $T_{U}$, we use an opposite approach than the one used for the $T_{L}$. Instead of using the standard (left-tailed) conditional cumulative probability, we calculate the right-tailed conditional cumulative probability. This involves starting from the highest possible response value (i.e., 3) and accumulating probabilities moving towards the lowest (i.e., 0). This method reflects the likelihood that a respondent, given their response history, will provide a score greater than or equal to a certain threshold. Also, the calculation is based on the probability that a respondent is non-depressed. The goal is to ensure that if we set an upper threshold at a particular response value, there is a low chance that a non-depressed individual would give such a high score. This allows us to classify respondents as depressed if their answer exceeds this threshold. We estimate the right-tailed conditional cumulative probability that a respondent is non-depressed (non−D), given their response history $H=\left(r_{1},r_{2},\ldots ,r_{i}\right)$ up to the previous question (i). For the next PHQ question ${\text{phq}}_{i+1}$, this probability is calculated as:

(2) $$P\left(non-D,phq_{i+1}\geq r|H\right)=\frac{P\left(non-D,phq_{i+1}\geq r,H\right)}{P\left(H\right)}$$

As with the lower threshold estimation, this calculation yields four cumulative probabilities corresponding to the four ordinal response values (0 to 3). The $T_{U}$ is determined using the following decision rule:

$$T_{U}=max\left\{\begin{array}{cc} 3\; & \text{if}p\left(non-D,phq_{i+1}\geq 3|H\right)>\lambda \\ 2\; & \text{if}p\left(non-D,phq_{i+1}\geq 2|H\right)>\lambda \\ 1\; & \text{if}p\left(non-D,phq_{i+1}\geq 1|H\right)>\lambda \\ 0\; & \text{if}p\left(non-D,phq_{i+1}\geq 0|H\right)>\lambda \;\;\text{or}\;P\left(non-D|H\right)=0 \end{array}\right)$$

#### 3.2.2 Thresholds estimation for phq1

Since phq1 has no prior responses, we estimate thresholds based solely on observed response distributions. First, we compute the probability cumulative distribution of depressed (D) for each response (r∈{0,1,2,3}). The lower threshold ($T_{L}$) then is determined as follow:

$$T_{L}=min\left\{\begin{array}{cc} 0\; & \text{if}P\left(D,phq_{1}\leq 0\right)>\lambda \\ 1\; & \text{if}P\left(D,phq_{1}\leq 1\right)>\lambda \\ 2\; & \text{if}P\left(D,phq_{1}\leq 2\right)>\lambda \\ 3\; & \text{if}P\left(D,phq_{1}\leq 3\right)>\lambda \end{array}\right)$$

Similarly, the upper threshold ($T_{U}$) is determined using the non-depressed group, but starting from the highest response value:

$$T_{U}=max\left\{\begin{array}{cc} 3\; & \text{if}p\left(non-D,phq_{1}\geq 3\right)>\lambda \\ 2\; & \text{if}p\left(non-D,phq_{1}\geq 2\right)>\lambda \\ 1\; & \text{if}p\left(non-D,phq_{1}\geq 1\right)>\lambda \\ 0\; & \text{if}p\left(non-D,phq_{1}\geq 0\right)>\lambda \end{array}\right)$$

#### 3.2.3 Handling sparse data using K-nearest neighbours (KNN)

In some instances, the conditional cumulative probability approach may result in an empty sample set due to the unique response patterns of certain individuals. In such cases, we apply density estimation using KNN to estimate thresholds. The input to the KNN model is the individual’s past questions. The output is the estimated threshold for the next question. The distance metric $d\left(X_{i},X_{j}\right)$ used for this estimation is the standard Euclidean distance as defined in Eq. (3), ensuring that the closest response patterns are identified for comparison.

(3) $$d\left(X_{i},X_{j}\right)=\sqrt{\overset{N}{\underset{n=1}{\sum }}{\left(X_{i}{,}_{n}-X_{j}{,}_{n}\right)}^{2}}$$

where:

• $X_{i}{,}_{n}$ and $X_{j}{,}_{n}$ are the responses of individuals i and j to the nth question in the PHQ assessment.
• N is the number of previous responses considered.

This distance function ensures that individuals with similar response patterns are grouped together, enabling an accurate estimation of the next threshold. Similarly to previous cases, the dataset is divided into depressed and non-depressed groups. However, for estimating the lower threshold ($T_{L}$), we focus exclusively on the non-depressed group. This strategy allows us to identify the most similar individual who is confirmed to be non-depressed and use their response as a safe estimate. Conversely, the upper threshold ($T_{U}$) is estimated using the depressed group to reflect the response behaviour of individuals who are truly experiencing depressive symptoms.

#### 3.2.4 Model hyper parameters

The model’s performance was optimized by varying five hyper parameters: the zero count parameter (ranging from 0 to 9), two parameters for the starting points for the classification (ranging from 0 to 3), the number of neighbours (K) in the KNN model (ranging from 1 to 4), and the λ parameter (ranging from 0.01 to 0.1). In clinical settings, the value of λ can be adjusted based on the desired risk tolerance. For simplicity, our model uses a single optimal λ value for estimating both the lower and upper thresholds. However, in practice, separate λ values could be used for each threshold to better tailor the model’s sensitivity and specificity, potentially improving diagnostic performance.

The optimal values for all hyper parameters were selected based on two criteria: maximizing the Youden index and minimizing the average number of questions required. The Youden index was chosen as a performance metric because it provides a balanced assessment of diagnostic accuracy by considering both sensitivity and specificity. This ensures that the selected threshold effectively identifies true positives while minimizing false positives, making it particularly suitable for evaluating screening models.

(4) Youden Index=Sensitivity+Specificity−1

Eq. (4) shows the formula for calculating the Youden index. To benchmark the dynamic approach, its results were compared against the PHQ-DEP-4. To ensure a fair comparison, the optimal threshold for distinguishing between positive and negative cases in the PHQ-DEP-4 was set at 4 for both datasets, allowing this reference standard to perform at its best.

## 4 Results

After running multiple iterations of the model to determine the optimal parameter values, the results consistently indicated that a zero count value of 6 produced the highest performance and the lowest average number of questions across all models, for both the NHANES and PROACTIVE datasets. The optimal value for the λ parameter was found to be 0.02 for both datasets. As for KNN, we experimented with multiple values for the k parameter and found that setting K = 1 yielded the best results for both datasets. This choice ensures that the threshold estimation is based on the most similar individual in the datasets. Similarly, for the initial classification parameters, the best performance, along with the lowest average number of questions was achieved when positive cases were allowed to be classified starting from the third question (phq3), while negative cases could be classified beginning from the second question (phq2) in both datasets. The detailed performance metrics for these optimal configurations are presented in Table 4.

**Fig. 2:**
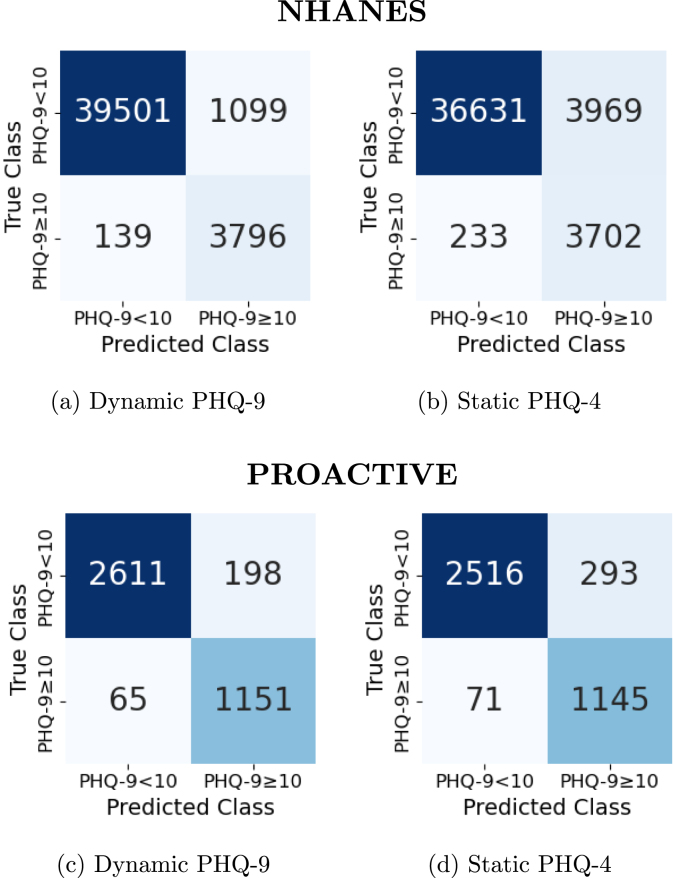
Confusion Matrices.

The Dynamic PHQ-9 model demonstrated the highest discrimination ability, achieving a Youden Index of 0.94, along with high sensitivity (0.96) and specificity (0.97) in the NHANES dataset. It also attained the highest accuracy (0.97) while requiring an average of 3.43 questions. Similarly, in the PROACTIVE dataset, the Dynamic PHQ-9 model achieved the highest Youden Index of 0.90, with sensitivity and specificity both at 0.95, and the highest accuracy of 0.93. Fig. 2 illustrates these results in terms of correct and incorrect classifications.

**Table 4:** Results.

Dataset	Model’s Name	Youden Index	Sensitivity	Specificity	Accuracy	Questions number (Avg)
NHANES	Dynamic PHQ-9	0.94	0.96	0.97	0.97	3.43
	Static PHQ-DEP-4	0.82	0.85	0.96	0.95	4
PROACTIVE	Dynamic PHQ-9	0.90	0.95	0.95	0.93	3.98
	Static PHQ-DEP-4	0.84	0.94	0.89	0.87	4

Fig. 2(a) shows that in the NHANES dataset, the Dynamic PHQ-9 model misclassified 1099 individuals as depressed, significantly fewer than the 3969 misclassified individuals in the static model (Fig. 2(b)). A similar pattern is observed in the PROACTIVE dataset, where the Dynamic PHQ-9 model misclassified 198 individuals as depressed (Fig. 2(c)), compared to 293 in the static model (Fig. 2(d)). Additionally, in both datasets, the Dynamic model outperformed the static model in correctly identifying non-depressed individuals, misclassifying 139 and 65 individuals in NHANES and PROACTIVE, respectively. In contrast, the static model misclassified 233 and 71 individuals in NHANES and PROACTIVE, respectively.

Moreover, in the NHANES dataset, the Dynamic PHQ-9 model maintained strong performance while requiring an average of 3.46 questions. The Static PHQ-DEP-4 model also performed well, requiring an average of 4 questions, but did not achieve the same level of performance as the Dynamic PHQ-9. In the PROACTIVE dataset, the Dynamic PHQ-9 model required an average of 3.98 questions, highly close to the static model. However, the Dynamic model outperformed the static model in terms of accuracy and classification efficiency.

**Fig. 3:**
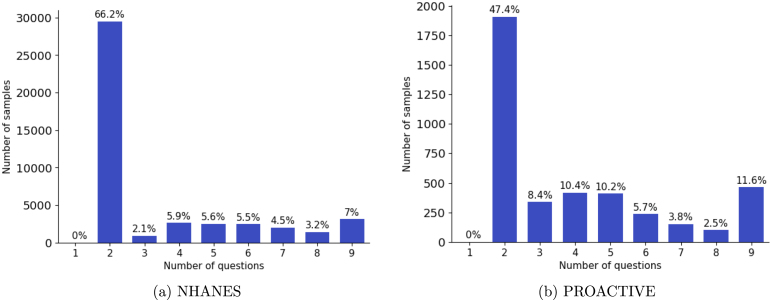
Histogram Shows Number of Cases Classified vs. Number of Questions Administered for Dynamic Models.

Fig. 3 presents histograms showing the number of individuals diagnosed at each question level for the Dynamic PHQ-9 models. Notably, in both datasets, no individuals were diagnosed after the first question (phq1). This is expected, as both Dynamic models initiated classification from the second question (phq2) for negative cases, as explained earlier in this section.

In the NHANES dataset (Fig. 3(a)), the majority of individuals (66.2%) were classified after answering only two questions (phq1 and phq2). A similar trend is observed in the PROACTIVE dataset (Fig. 3(b)), where 47.4% of individuals were diagnosed after two questions. Additionally, in NHANES, only 7% of participants needed to complete all nine PHQ-9 questions, compared to 11.6% in the PROACTIVE dataset.

**Fig. 4:**
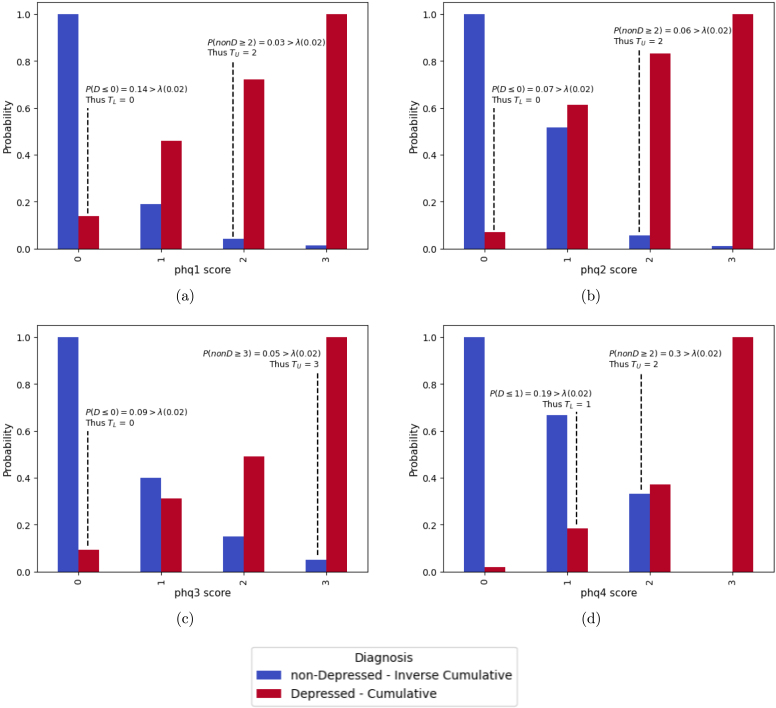
Example shows how the upper and lower thresholds are selected for a specific respondent based on their answers.

Fig. 4 presents an illustrative case from the NHANES dataset involving a respondent who was ultimately diagnosed as depressed. The figure demonstrates how the upper and lower thresholds were estimated across the first four PHQ-9 items, eventually leading to a correct classification by the fourth question.

In each subfigure, red bars represent individuals whose total PHQ-9 score is ≥10, indicating depressive symptomatology and who where hence labelled as depressed. Blue bars represent individuals with PHQ-9 scores <10, corresponding to the non-depressed population.

In Subfigure (a), we estimate the lower threshold ($T_{L}$) using the cumulative probability distribution of the depressed group (red bars). All possible phq1 scores (0, 1, 2, 3) yield cumulative probabilities above the predefined λ threshold. Since we aim to identify non-depressed individuals as early as possible, we select the lowest score that meets the criterion, which is (0). Therefore, the lower threshold for phq1 is set to (0), meaning if the respondent answers (0), they would be classified as non-depressed.

For the upper threshold ($T_{U}$), we use the cumulative probability distribution of the non-depressed group (blue bars). This approach helps identify individuals with high early responses who are likely to be depressed. We search from the highest possible score downward. in this case, the cumulative probability at score (3) is below λ, so it does not satisfy the condition. However, scores (2, 1, and 0) do exceed λ, and we choose (2) as the highest threshold that meets the condition. This implies that if the respondent answers >2, they are classified as depressed. In Subfigures (b), (c), and (d), the same process is applied, but using conditional cumulative probabilities. Specifically, we only consider data points whose previous answers match the current individual’s response pattern. This conditioning ensures that threshold estimates are based on behaviourally similar cases, improving the accuracy and efficiency of early diagnosis. For this case, the final estimated thresholds across the first four PHQ items are:

• Lower thresholds: [0, 0, 0, 1].
• Upper thresholds: [2, 2, 3, 2].

We apply cumulative sums to both the thresholds and the respondent’s answers to make classification decisions after each question. In this example this yields:

• Cumulative lower thresholds: [0, 0, 0, 1].
• Cumulative upper thresholds: [2, 4, 7, 9].

At each step, the model compares the cumulative score of the respondent to these thresholds. If it is ≤ the cumulative $T_{L}$, the respondent is classified as non-depressed; if it is > the cumulative $T_{U}$, they are classified as depressed. Otherwise, the model continues to the next question.

**Fig. 5:**
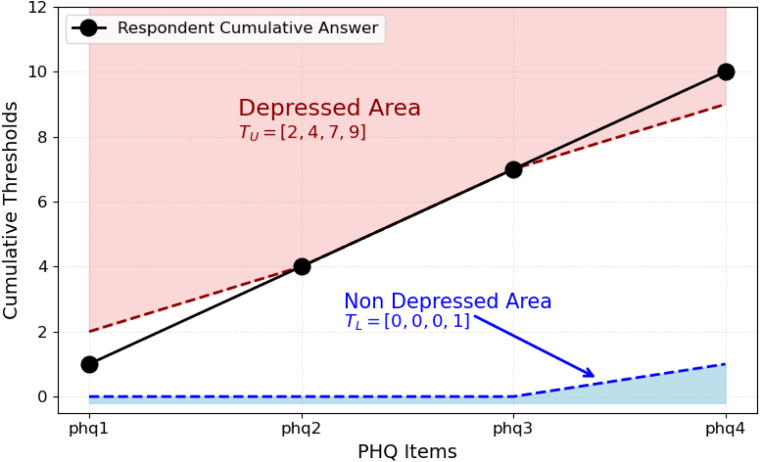
A Single Case to Show the Decision Boundary Visualization Using Cumulative Thresholds.

Additionally, Fig. 5 displays the decision boundary plot for this specific case, using the cumulative upper and lower thresholds. The plot is divided into three distinct regions defined by these thresholds. The red area represents the depressed region, any cumulative scores falling within this zone result in a classification of depression. Conversely, the blue area indicates the non-depressed region, where scores lead to a non-depressed classification. The white area between these two represents the neutral zone, where the model requires additional information (i.e., another question) before making a final diagnosis.

In the plot, the respondent first undergoes evaluation after (phq1), and their cumulative score falls within the white area, indicating the need for another question. After answering (phq2), their cumulative score approaches the boundary of the depressed region but still falls within the neutral zone, prompting further questioning. This pattern continues at (phq3). Finally, at (phq4), the cumulative score surpasses the cumulative upper threshold and lands in the red area. At this point, the model classifies the respondent as depressed, and the assessment process is terminated.

## 5 Discussion

One significant limitation of this study is its reliance on the PHQ-9 total score as the ground truth for distinguishing between depressed and non-depressed individuals. While this is a widely accepted approach in research, it does not replace clinical diagnosis by a healthcare professional. The PHQ-9 is a self-reported measure and may not capture the full complexity of an individual’s mental health status. Future research should evaluate the model’s performance against clinician-administered diagnostic interviews, such as the Structured Clinical Interview for DSM (SCID), to determine its real-world diagnostic accuracy.

It is well established that the prevalence of depression varies across countries, cultures, and societies. Interestingly, our dynamic model demonstrated consistently high and very similar performance across both datasets NHANES and PROACTIVE, despite a substantial difference in depression prevalence (8.8% in NHANES vs. 30% in PROACTIVE). The difference in key performance metrics between the two datasets was minimal (NHANES: sensitivity 0.96, specificity 0.97; PROACTIVE: sensitivity 0.95, specificity 0.95), suggesting that the model is robust and performs reliably even when applied to populations with markedly different baseline rates of depression. This indicates that prevalence alone may not significantly impact the model’s effectiveness. However, to further investigate this relationship, additional testing on datasets from other countries with varying prevalence rates would be valuable. Such analysis would help confirm the model’s generalizability, consistency, and reliability in distinguishing between depressed and non-depressed individuals across diverse populations.

Furthermore, higher variability in a dataset may actually enhance the model’s ability to classify cases accurately. Since the model relies heavily on conditional probability, it effectively filters for similar response patterns to estimate thresholds. Greater diversity in the dataset increases the likelihood of having enough comparable cases to support confident classification. In this way, dataset variability could improve the model’s diagnostic capability.

## 6 Conclusion

This study introduces a dynamic model that adapts the PHQ-9 depression screening process, offering an alternative to fixed-length versions such as the PHQ-Dep-4. The dynamic model, in which the number of questions administered varies according to the respondent’s answers. This dynamic approach uses lower and upper cumulative threshold that are estimated using the probability of depression conditional on the responses given thus far and can terminate the assessment early when a confident classification (depressed or non-depressed) can be made before all nine questions are completed. The dynamic model relies on a historical datasets of completed PHQ-9 interviews to inform these decisions. When a matching response pattern is not available in the historical data, a KNN model is applied to estimate the conditional probabilities used to come to decisions. Five key hyperparameters govern the dynamic model: the zero-count, Lambda (represents the acceptable risk of misclassifying), two parameters for the starting points for positive and negative cases, and the number of neighbours (K) in the KNN model. These parameters were tuned to maximize the Youden index while minimizing the number of questions asked.

The dynamic model was tested using two distinct datasets: NHANES from the U.S. and PROACTIVE from Brazil, representing different populations and healthcare environments. Compared against the PHQ-DEP-4, the dynamic model consistently demonstrated higher performance. In the NHANES dataset, the dynamic model achieved a Youden index of 0.94, along with high sensitivity (0.96) and specificity (0.97). Similarly, in the PROACTIVE dataset, the dynamic model achieved a Youden index of 0.90, with sensitivity and specificity both at 0.95. These results highlight the model’s potential as an effective and efficient screening tool for depression.

Beyond improving classification accuracy, the dynamic model also reduces respondent burden. In NHANES, an average of just 3.46 questions were required to reach a diagnosis; in PROACTIVE, the average was 3.98, both outperforming PHQ-DEP-4 while using fewer questions. This makes the dynamic model an efficient and effective alternative to static screening tools.

The dynamic model effectively identifies depressed and non-depressed individuals early in the assessment process, demonstrating potential to enhance mental health screening by reducing respondent burden while maintaining diagnostic reliability. Future research should investigate its applicability across broader populations and validate its performance with clinical diagnoses to further establish its effectiveness.

## Code availability

The Python implementation of the personalized PHQ-9 model developed in this study is publicly available on GitHub at:

https://github.com/zahraa-m/Personalised-PHQ-9-Model.

A permanent archived version of the code is available via Zenodo with the following DOI:

https://doi.org/10.5281/zenodo.18623952.

## Funding

This work has emanated from research conducted with the financial support of 10.13039/501100001602Research Ireland under Grant Number SFI 18/CRT/6049.

## Declaration of competing interest

The authors declare no conflicts of interest.
